# Analysis and removal of bisphenols in recycled plastics using polyethylene glycol

**DOI:** 10.1038/s41598-024-63800-7

**Published:** 2024-06-04

**Authors:** Samuel S. Núñez, Núria Ortuño, Sabrina Fernández-Durán, Julia Moltó, Juan A. Conesa

**Affiliations:** 1 Department of Chemical Engineering, University of Alicante, P.O. Box 99, 03080 Alicante, Spain; 2 Institute of Chemical Process Engineering, University of Alicante, P.O. Box 99, 03080 Alicante, Spain

**Keywords:** Environmental chemistry, Environmental impact

## Abstract

This study examines the presence of bisphenol A (BPA), S (BPS), F (BPF), and M (BPM) in various recycled plastics readily available on the market (LDPE, HDPE, PET, and PP), in light of European Food Safety Authority (EFSA) limits. Twenty samples of different origin are analyzed, cleaning treatments are applied, and the migration potential of these bisphenols into food is studied. BPM is absent in all samples, but a post-consumer recycled LDPE sample reveals high bisphenol concentrations, raising concerns, reaching 8540 ng/g, 370 ng/g, and 29 ng/g of BPA, BPS, and BPF, respectively. Migration tests show substantial migration of these contaminants into food simulants. Using a cleaning treatment with polyethylene glycol (PEG 400) reduces BPA in LDPE, HDPE, PP, and PET samples by 95%, 99%, 97% and 28%, respectively, highlighting the importance of cleaning treatments across various polymers in plastic recycling. These findings not only protect food safety but addressing environmental challenges associated with plastic recycling.

## Introduction

Plastics, valued for their strength and adaptability, have proliferated, yet their extensive use contributes to environmental challenges like ecosystem pollution and greenhouse gas emissions. Responsible action is essential for fostering a more sustainable society. To address this, understanding global and regional dynamics in plastics production and consumption is crucial. According to Plastics Europe^1^, global plastics production rose by 2.4% in 2022 to 400.3 million tonnes, predominantly sourced from petroleum-based materials. Europe, ranking third in production with 58.7 million tonnes, faces similar challenges in managing plastics, notably in packaging, which comprises 39% of global production.

Despite these challenges, signs of progress in plastics recycling are evident. In Europe, 12.9% of plastic produced in 2022 was recycled, marking a 2.8% increase from the previous year^1^. Innovative technologies and strategies are emerging to mitigate plastic consumption, foster reuse and recycling, and curb environmental dispersion. Initiatives such as eco-design and the circular economy advocate for resource prevention, reduction, repair, reuse, and recycling^2–4^. Efforts are underway to boost recycled plastics utilization, with a targeted 55% plastic recycling rate by 2030^5^.

One of the primary challenges in plastic recycling is the risk of chemical substances migrating into food products, affecting their safety and quality^6^. These substances fall into two categories: Intentionally Added Substances (IAS) and Non-Intentionally Added Substances (NIAS).

IAS are deliberately incorporated into plastics for specific purposes, such as enhancing mechanical, thermal, or antimicrobial properties^7^. Examples include additives, dyes, plasticizers, and stabilizers. These substances must comply with regulations, such as Regulation (EU) No. 10/2011, which establishes a positive list of authorized IAS, along with their overall migration limit and specific migration limit^8^.

On the other hand, NIAS are substances that are not deliberately added to plastic but originate from various sources, such as the degradation or decomposition of IAS, impurities in raw materials, contamination during the manufacturing or recycling processes, or reactions between plastic components or with the environment. It is important to note that some NIAS could be present in the positive list of Regulation 10/2011/EU due to cross-contamination, even if the substances have not been intentionally added. Additionally, Regulation (EC) No. 1935/2004 applies to all substances, not just NIAS, so they must also be evaluated according to the general principle established in Article 3 of Regulation (EC) No. 1935/2004 on materials and articles intended to come into contact with food^9^, which states that these substances must not endanger human health or alter the composition or organoleptic characteristics of food products.

Some examples of NIAS include oxidation products, oligomers, residual catalysts, or environmental contaminants. Among the latter, persistent organic pollutants (POPs) such as polycyclic aromatic hydrocarbons (PAHs) and polychlorinated dibenzo-*p*-dioxin and dibenzofurans (PCDD/Fs) have been detected in samples of recycled low-density polyethylene (LDPE) plastics from various sources, including post-industrial, and post-consumer plastics. While it is uncommon to find dioxins in this type of plastics, scientific literature documents cases where they have been detected^10^. Additionally, more than 300 substances were found in a migration test on post-consumer plastic samples (HDPE flakes and pellets from milk bottles), with 58 substances classified as toxic^11^.

In this context of concern for food safety and the quality of materials in contact with food, the detection and evaluation of substances such as bisphenols in recycled plastics become critical aspects of scientific research, particularly because studies, such as the one conducted by Dreolin et al.^12^ have already demonstrated the presence of this compound in recycled plastics, specifically in recycled PET. Furthermore, other authors revealed that as the amount of recycled PET increases, the quantity of BPA in beverages significantly rises, highlighting concerns regarding the safety of recycled PET^1^^1,3^^4^.

Bisphenol A (BPA) is known for its potential health impacts and is found in various consumer products^15^. Concerns about BPA have led to the development of substitutes like Bisphenol F (BPF) and Bisphenol S (BPS), but research suggests these may have similar health effects ^16^. While studies on BPS and BPF are limited, their mechanisms resemble BPA^17^. Bisphenol AF (BPAF) and Bisphenol M (BPM) may be more toxic than BPA, highlighting the need for further research and regulation^18^. Regulations on BPA have tightened over time, with the EU imposing restrictions on its use, especially in infant products, and lowering migration limits for food contact materials. In 2023, the European Food Safety Authority (EFSA) established a much lower tolerable daily intake for BPA^19^. Despite efforts, BPA and its analogues remain concerning due to their widespread presence and potential health effects. Continued regulation and research are crucial to address these risks and ensure public and environmental safety.

In this context, recycling plays a pivotal role in mitigating the negative impacts of plastic production and waste by offering a sustainable solution. However, persistent challenges, particularly concerning the presence of chemical substances in recycled plastics, remain. As we transition towards a circular economy, achieving higher recycling rates and ensuring the safety and quality of recycled materials are imperative. This is especially crucial when considering substances like BPA and its analogues in recycled plastics, highlighting the necessity for effective management of these contaminants to promote responsible recycling practices. While bisphenols are typically added as additives to polycarbonates^19^, they can also be present as NIAS in other plastics. Therefore, it is crucial to examine the presence of Bisphenol A (BPA) in plastics such as LDPE, HDPE, PP, and PET^20^.

For this reason, before attempting to remove contaminants through appropriate treatment during the recycling process, it is imperative to comprehend the potential migration of these compounds from recycled plastics (pellets) into final products, particularly those intended for contact with food. Such migration could have critical implications for food safety and human health. Our research also focuses on analyzing the migration of chemical contaminants in recycled plastics, adhering to European Union Regulation 10/2011^8^. These tests are conducted using two distinct food simulants and ultrapure water. While historically such migration tests were applied to establish criteria for decontamination efficiency, our aim is to assess the impact of these contaminants on food quality and safety. Additionally, Hoekstra et al.^21^ provided critical insights into BPA migration from polycarbonate to various food simulants under diverse conditions. Furthermore, Wong et al.^22^ achieved up to 0.19 mg/L BPA using 10% ethanol simulant 10 day at 65 °C. Moreover, Guart et al.^23^ conducted a migration test on various plastics, revealing that BPA migrated from HDPE and LDPE caps. This will enable an assessment of its impact on food quality and safety.

The responsible management of chemical contamination in recycled plastics is crucial for ensuring end product safety and human health. Thus, evaluating the effectiveness of cleaning treatments, our research focuses on an innovative approach utilizing polyethylene glycol (PEG) to remove chemical contaminants, including bisphenols, with the goal of achieving nearly complete removal. PEG's versatility, as a low molecular weight polymer, non-volatile solvent with high solubility in polar solvents like water^24^, along with its biodegradability, biocompatibility, and non-toxicity, makes it a safe and environmentally friendly option. This approach has the potential to revolutionize the plastic recycling industry and improve product quality.

Cabanes et al.^25^ demonstrated PEG's effectiveness in removing volatile organic compounds from post-consumer high-density polyethylene (HDPE) samples at 100 °C, resulting in a substantial 70% reduction in concentration. Núñez et al.^26^ furthered this research, achieving significant reductions of 89%, 85%, and an impressive 95% in Polycyclic Aromatic Hydrocarbons (PAHs), Polychlorinated Dibenzo-p-Dioxins and Dibenzofurans (PCDD/Fs), and dioxin-like Polychlorinated Biphenyls (PCBs), respectively, in recycled low-density polyethylene (LDPE) samples. Zhou et al.^27^ studied PEG 200-water solution's efficacy in extracting organic compounds from plants, showing increased extraction compared to pure water. Alanazi et al.^28^ demonstrated the solubility of organic molecules in PEG 400 despite low solubility in water. Additionally, Kianpour and Azizian^29^ achieved 98% reductions in compounds from liquid fuels using PEG. These studies highlight PEG's versatility in addressing various organic contaminants, including BPA, BPS, BPM, and BPF, underscoring its value as a tool in addressing organic contaminants.

Despite PEG treatment shows promise in removing contaminants from recycled plastics, understanding the fate of glycols post-treatment and their potential migration impact is crucial for ensuring end product safety and quality. Additionally, while PEG is versatile in removing organic contaminants from recycled plastics, it's essential to note that polyolefins like LDPE, HDPE, and PP are not authorized for food contact materials. This restriction stems from the fact that while the treatment may target specific contaminants, it may not eliminate other unmentioned compounds.

In conclusion, it is crucial to present the hypothesis of this research. It is proposed that the concentrations of bisphenols (A, F, S, M) will vary significantly among different types of recycled plastics (LDPE, HDPE, PP, PET). Additionally, it is anticipated that the migration of bisphenols in food simulants will also vary depending on the type of recycled plastic. Finally, it is hypothesized that the decontamination treatment with polyethylene glycol will result in substantial reductions of bisphenols in all samples of recycled plastics.

## Results and discussion

### Bisphenols content in recycled and virgin plastic samples

**Figure 1:**
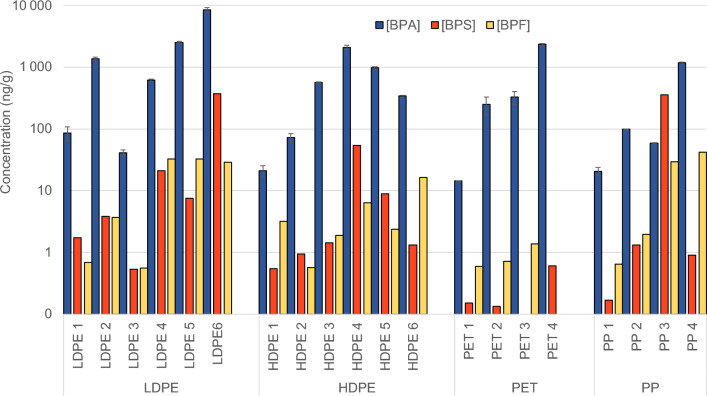
Concentrations of bisphenols (BPA, BPS and BPF) in the various recycled and virgin plastic.

In Fig. 1, the results obtained for the concentrations of BPA, BPS, and BPF in recycled plastics available in the market are depicted, categorized by the type of contaminant analyzed for all plastic samples used. Samples LDPE1, HDPE1, PET1 and PP1 correspond to samples of virgin materials of each plastic type. While the presence of BPA, BPS and BPF in the results has been confirmed, substantiating concerns regarding potential exposure to these compounds known for their adverse effects on human health and the environment, it is noteworthy that, despite rigorous analytical methodology and the use of various plastic types, no presence of BPM has been detected in any of them.

The absence of BPM in the samples could be attributed to its low prevalence compared to the other analyzed bisphenols. In fact, in a previous study that comprised the analysis of various bisphenols, including those investigated in our study, BPM was detected in 75% of the samples. However, the concentrations of BPM were found to be significantly lower, with ratios 103, 9, and 5 times lower than those of BPA, BPS, and BPF, respectively^30^. Nonetheless, for a comprehensive understanding of the lack of BPM detection in these samples, additional investigations are required to explore the dynamics of bisphenols during plastic recycling and their impact on the presence or absence of BPM.

Continuing with the results, Table 1 shows the results of the analysis of Bisphenols A, S and F in various plastic types, including LDPE, HDPE, PET, and PP, in both virgin and recycled plastics. Furthermore, in Fig. 1, the concentrations of the bisphenols are shown. The results unveiled substantial variability in BPA concentrations within the analyzed samples, shedding on the complexity of this issue.

**Table 1:** Concentrations (mean and standard deviation, SD, in ng/g) of bisphenols A, S and F (BPA, BPS and BPF respectively) in the different samples.

Samples	Concentrations (ng/g)					
	[BPA]	SD	[BPS]	SD	[BPF]	SD
LDPE						
LDPE 1	86.3	22.4	1.72	0.22	0.69	0.24
LDPE 2	1380	72.3	3.84	0.77	3.71	0.34
LDPE 3	41.2	4.46	0.54	0.09	0.56	0.18
LDPE 4	623	25.3	21.1	10.7	32.9	7.21
LDPE 5	2530	82.9	7.57	1.76	33	6.47
LDPE6	8540	667	372	4	29.1	8.62
HDPE						
HDPE 1	21	4.18	0.55	0.15	3.16	1.12
HDPE 2	72.5	12.4	0.95	0.36	0.56	0.03
HDPE 3	569	8.69	1.44	0.08	1.88	0.7
HDPE 4	2110	144	54.2	16.8	6.36	3.08
HDPE 5	978	40.3	8.89	1.54	2.36	0.33
HDPE 6	340	11.3	1.32	0.32	16.2	3.08
PET						
PET 1	14.5	0.05	0.15	0.03	0.59	0.07
PET 2	251	81.7	0.13	0.03	0.72	0.15
PET 3	326	79.9	0.00	0.00	1.38	0.25
PET 4	2350	84.8	0.61	0.09	0.00	0.00
PP						
PP 1	20.8	2.96	0.17	0.01	0.64	0.25
PP 2	99	0.2	1.31	0.23	1.98	0.07
PP 3	59.1	0.9	361	35.5	29.3	5.42
PP 4	1170	36.7	0.9	0.3	41.8	0.97

One of the findings was the detection of generally low concentrations of BPA in virgin plastic samples (e.g., LDPE1, HDPE1, PET1, and PP1), ranging from 10 to 90 ng/g. These results suggest that a significant portion of the BPA found in the remaining samples may be classified as Non-Intentionally Added Substances (NIAS), introduced during the plastic's lifecycle and accumulation in recycling facilities. This is evident when comparing the BPA levels in virgin plastic with recycled plastic, as recycled plastic contains higher concentration. If BPA were intentionally added, higher levels would be expected in the virgin plastic.

The importance of washing in reducing BPA contamination in recycled plastics was clearly emphasized in this study. For instance, LDPE 3, a recyclate subjected to a patented special washing process, exhibited a concentration of 40 ng/g, lower than that found in virgin plastic (86 ng/g). This underscores the effectiveness of washing techniques in removing contaminants, with significant implications for improving the quality of recycled plastics.

However, an important observation was highlighted in PET samples 2 and 3, representing the input and output of a pre-recycling cleaning process. Contrary to expectations, PET 3 showed a slightly higher BPA concentration than PET 2 after the cleaning process, with concentrations of 320 ng/g and 250 ng/g, respectively. This could be attributed to various factors such as the quality of the washing water or the challenge of BPA removal from this type of plastic, emphasizing the need for further research to better understand these processes. PET 4 was the most contaminated in BPA within PET samples. This material consists of recycled PET filament for use in 3D printing, and the details on the recycling procedure are unknown to the authors.

The color of the plastic may also play a role in BPA concentration, as it is commonly added using a masterbatch. This masterbatch often contains a high concentration of dye and compatibilizers (a blend of chemicals), which could contribute to the presence of bisphenols and other compounds. Bamfield^31^ highlights that despite its activity as an endocrine disruptor, bisphenol A (BPA) is extensively employed in the manufacturing process as a dye or pigment due to its capability to produce vivid hues and enhance contrasts. This suggests a plausible association between the coloration of plastics and the presence of BPA, warranting further investigation into the potential implications of this relationship. Another more recent study suggests that bisphenol A (BPA) may be a common component in thermochromic printing inks, and the precise composition of these inks is often not disclosed due to patent protection ^32^.

An example of this is that post-industrial LDPE samples LDPE 4 and LDPE 5 showed significant differences in BPA concentrations, with LDPE 5 (black) exhibiting concentrations exceeding 2500 ng/g, while LDPE 4 (white) remained around 600 ng/g. A similar pattern was observed in polypropylene samples PP 3 and PP 4, which, despite having the same origin and treatment, had very different concentrations; specifically, PP 4 (black) had a concentration 20 times higher than PP 3 (green). This suggests the need for additional research that incorporates samples of different colors to assess the influence of color on BPA absorption.

Furthermore, recycling conditions and the type of recycled waste appear to influence BPA concentrations. For example, samples HDPE 2 and HDPE 3, originating from different regions and subjected to variable recycling conditions, showed significant differences in BPA concentrations, ranging from 70 to 570 ng/g. These results underline the importance of proper management of recycled plastics to minimize environmental exposure to BPA.

Regarding Bisphenol S (BPS) (also shown in Fig. 1), as with BPA, it is essential to note that virgin plastic samples (LDPE 1, HDPE 1, PET 1, and PP 1) exhibit some of the lowest concentrations of BPS. Consequently, it could also be inferred that BPS is a substance not intentionally added in the samples of recycled plastic.

Once again, the importance of the washing process during recycling in reducing BPS concentrations in recycled plastics is highlighted. LDPE 3, derived from recycled plastic and subjected to a rigorous washing process, shows significantly lower concentrations of BPS compared to other recycled LDPE samples. Furthermore, samples PET 2 and PET 3, representing the same material before and after the cleaning process, also exhibit a reduction in BPS concentrations after washing (0.1 ng/g and not detected respectively). These findings indicate that washing can also play a crucial role in decreasing the presence of BPS in recycled plastics. HDPE 2 and PP 2 also show low BPS concentrations in their respective groups, specifically 0.95 and 1.31 ng/g, which may be influenced by their washing with hot water.

When considering color, the concentration of BPS exhibits exactly the opposite trend to that of BPA, with the concentration of LDPE 4 (white) being nearly 3 times higher than that of LDPE 5 (black), and the concentration of PP 3 (green) being the highest found in the polypropylene group, reaching a value of 360 ng/g.

To conclude the discussion of bisphenol concentrations in plastic samples, we address the concentrations of bisphenol F (BPF), also shown in Fig. 1.

As in the previous cases, a consistent pattern emerges where virgin samples consistently exhibit lower concentrations of BPF compared to recycled samples. It is noteworthy that both manufacturing processes and quality standards regarding the production of virgin plastics are stringent, resulting in reduced contamination by BPF. Additionally, LDPE 3, HDPE 2, and PP 2 samples showed very low concentrations in their respective groups, specifically 0.56 ng/g, 0.56 ng/g, and 1.98 ng/g, due to their previously mentioned cleaning processes.

As in the case of BPS, samples PET 2 (0.72 ng/g) and PET 3 (1.38 ng/g) showed little variation, even though PET 3 underwent the washing process before recycling.

Finally, in the case of BPF, color did not seem to influence the concentration as in the previous cases. LDPE 4 and LDPE 5 samples showed concentrations of 32.9 ng/g and 33.0 ng/g, respectively, and PP 3 and PP 4 exhibited concentrations of 29.3 ng/g and 41.8 ng/g.

In conclusion, the results underscore the complexity of bisphenol contamination in plastics. Generally low concentrations were found in virgin plastic, and washing was demonstrated to be effective in reducing concentrations in recycled plastics. However, there were exceptions, such as the increase in BPA concentrations in a PET sample after washing. Additionally, the color of the plastic appeared to be related to their bisphenol concentrations. These findings highlight the need for proper management of recycled plastics and the importance of further investigating these processes.

### Migration test results

In the context of this study, a comprehensive assessment of the migration of bisphenols (BPA, BPS, BPF, and BPM) from a post-consumer recycled plastic sample, was conducted. Specifically, post-consumer recycled low-density polyethylene (LDPE 6) was selected due to its higher concentration of BPA and BPS compared to all other samples analyzed (see Fig. 1).

**Figure 2:**
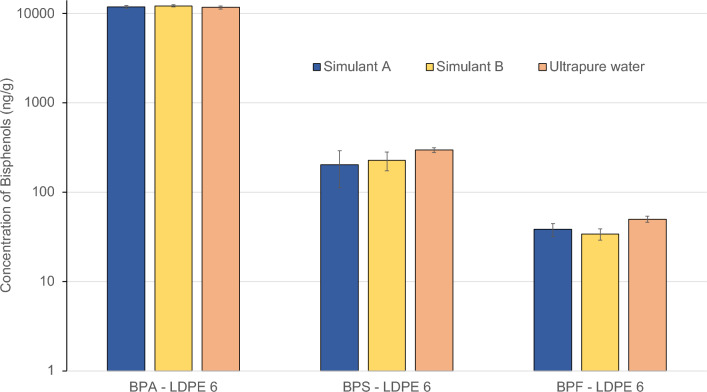
Concentrations of bisphenol A, bisphenol S, and bisphenol F in various food simulants and ultrapure water. Concentration in the simulant after 10 days of contact at 60 °C.

Plastic samples were subjected to migration tests consisting in the exposure of the recycled plastics to food simulants for 10 days at 60 °C, and the food simulants were analyzed for bisphenol content. The results of the pollutant concentrations in the migration test (expressed in nanograms of bisphenol per nanograms of plastic exposed to the simulant) are shown in Fig. 2. Note that the amounts are NOT expressed per mL of simulant.

In the analysis, it is pertinent to emphasize the absence of significant differences in the concentrations of the various bisphenols among the three food simulants employed (10% v/v ethanol in water for simulant A and 3% v/v acetic acid in water for simulant B, and ultrapure water for C), i.e., the migration from the plastic material is very similar for the three cases, as the detected concentrations fall within the same magnitude range for all two simulants and ultrapure water.

Furthermore, a noteworthy phenomenon is observed concerning Bisphenol A. The concentrations of BPA detected in the three food simulants substantially exceed the concentrations previously established in the plastic extraction process, which were 8540 ng/g. Specifically, concentrations of 11,800 ng/g, 12,200 ng/g, and 11,700 ng/g were recorded for food simulants A, B, and ultrapure water C, respectively. This increase could be attributed to the application of extreme conditions for the migration test (a 10-day contact period at 60 °C), which can cause the migration of the total amount of BPA.

In our results, we observed a BPA migration that reached 142% compared to the initial concentration. This observation, although surprising, is not unique in the scientific literature^33^, in a prior study using a 50% ethanol in water simulant under similar conditions, found BPA recovery percentages of up to 368%. While our research focused on recycled plastics rather than polycarbonate, these previous results highlight the variability in BPA migration under different conditions and underscore the need for further in-depth analysis. The observation cannot be conclusively explained within the scope of this study due to the lack of additional information about the underlying causes of this phenomenon.

Regarding BPS, it is observed that the concentrations recorded in the migration test remain slightly below the concentration determined in the extraction process. Similarly, BPF presents comparable concentrations. This observation may be related to the lower initial quantity of these contaminants in comparison to BPA, which could influence the migration rate. Finally, just as in the extraction process, BPM was not detected in any of the analyzed samples.

In conclusion, the findings of this study underscore the importance of carefully considering the migration process in post-consumer recycled plastic packaging when in contact with food and beverages. It is particularly significant to highlight the importance of thorough cleaning and decontamination stage during the recycling process to effectively remove contaminants, especially BPA, thus ensuring the safety of final products intended for human consumption. These findings emphasize the need for regulatory measures and stringent quality control in the plastic recycling industry to preserve public health and food safety.

### Cleaning treatment results

This section focused on assessing the effectiveness of a solvent-based cleaning treatment using polyethylene glycol (PEG 400) in reducing bisphenols (BPA, BPS, and BPF) in four common types of recycled plastics: LDPE, HDPE, PP, and PET. Independently of the results obtained, it is important to highlight that recycled LDPE, HDPE, and PP are not authorized for contact with food. Additionally, it is important to note that there are currently no accepted technologies for recycling and providing these decontaminated polymers.

**Figure 3:**
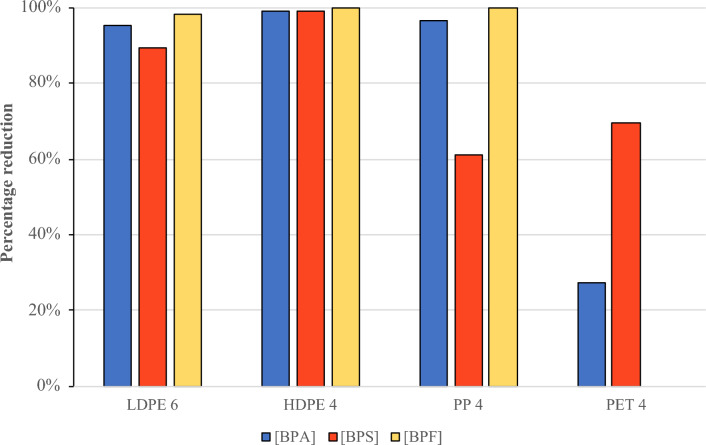
Evaluation of the efficacy of PEG 400 treatment in reducing contaminants in recycled plastics.

The most contaminated samples of each plastic type, totaling four, were selected to represent the recycling industry’s reality (LDPE 6, HDPE 4, PP 4, and PET 4). The results of the contaminant reduction percentages are presented in Fig. 3.

The LDPE sample exhibited promising outcomes, with significant reductions in BPA (95%), BPS (90%), and BPF (95%) following the treatment application. These figures suggest that LDPE is particularly responsive to PEG 400, which may be attributed to its permeability^34^, facilitating interactions with the solvent. When treating the HDPE sample, PEG demonstrated even greater effectiveness, achieving a 99% reduction in BPA and BPS, and complete elimination of BPF (100%). These results could be linked to the denser structure of HDPE, which likely allows for lower retention of bisphenols within its matrix, thereby facilitating the action of PEG 400.

However, the PP sample, despite achieving significant reduction in BPA (97%) and complete elimination of BPF, exhibited a less effective reduction in BPS, with a 61% decrease. This disparity may be attributed to the specific chemical properties of PP, which might not be as conductive interaction with PEG 400 in the case of BPS.

In the case of PET, the results are less promising, with lower reductions in BPA (28%) and BPS (70%). PET does not exhibit a reduction for BPF because this compound was not detected in the original sample of recycled PET, neither before nor after applying the cleaning treatment. The polarity of PET may influence its ability to retain certain compound that have some degree of polarity^35^, as in the case of BPA and BPS^36^, which could have led to limited efficacy in the treatment.

## Conclusions

The analysis conducted on Bisphenol A (BPA), Bisphenol S (BPS), and Bisphenol F (BPF) concentrations in plastic samples, both virgin and recycled, has unveiled significant variability, thus elucidating the intricate nature of bisphenol contamination in plastics. Notably, Bisphenol M (BPM) was undetectable in any of the samples, indicating its absence in both virgin and recycled plastics.

The analysis of virgin plastic samples showed low concentrations of BPA, BPS, and BPF, suggesting that much of these compounds in recycled samples might come from unintentional sources rather than intentional ones.

This study emphasizes the pivotal role of washing procedures in recycling for diminishing the presence of BPA, BPS, and BPF in plastics. Particularly, LDPE 3, subjected to a specialized washing process, exhibited reduced levels of these compounds compared to virgin plastic, illustrating the efficacy of washing techniques in enhancing recycled plastic quality. However, inconsistencies emerged as some washed samples, notably PET 2 and PET 3, showed increased BPA concentrations post-washing, raising apprehensions regarding PET's BPA removal efficacy and the quality of washing methods or water employed.

Furthermore, the study delved into the influence of color on bisphenol concentrations, revealing significant disparities in BPA levels among samples of the same origin but differing colors. This accentuates the necessity for further research to evaluate the impact of color or associated additives on bisphenol concentrations.

Regarding migration tests conducted on the most contaminated post-consumer recycled plastic sample (LDPE 6) using various food simulants and ultrapure water, notable findings emerged. No significant differences were discerned in bisphenol concentrations among the three food simulants, with concentrations akin to those observed in the original plastic. This persistent migration behavior, attributed to the utilization of extreme conditions in the migration test, underscores the imperative of meticulous cleaning and decontamination in plastic recycling processes to ensure food safety.

The efficacy of the cleaning treatment based on polyethylene glycol (PEG 400) extraction was also assessed, demonstrating considerable effectiveness in reducing bisphenols in LDPE and HDPE samples. However, diminished efficacy was observed in PP and PET samples, particularly when dealing with very low concentrations.

In summary, this study elucidates the presence of hazardous substances, such as BPA, BPS, and BPF, in varying concentrations in recycled plastics. It underscores the pivotal role of effective cleaning processes during recycling in reducing these substances, thereby ameliorating the quality of recycled plastics and averting potential hazards to food safety. However, discrepancies and exceptions underscore the necessity for stringent recycling practices, additional research, and regulatory measures to safeguard public health and ensure the safety of recycled plastics.

## Materials and methods

### Sample collection

The analysis in this study encompassed a diverse selection of polymers, including six samples of low-density polyethylene (LDPE), six samples of high-density polyethylene (HDPE), four samples of polypropylene (PP), and four samples of polyethylene terephthalate (PET). These polymers were obtained from various sources, including local recycling and consumer packaging. Detailed information regarding these various plastics, including their respective origins and characteristics, is presented in Table 2. This deliberate diversity in sample sourcing enabled a comprehensive assessment of treatment efficacy and facilitated an in-depth analysis of contamination and migration variability across distinct recycling contexts.

**Table 2:** Features of the plastic samples.

ID	Type	Subtype	Plastic Color	Treatment
LDPE 1	Virgin	Virgin	Transparent	None
LDPE 2	Post-Consumer	Agricultural	Brownish	Washed and re-extruded
LDPE 3	Post-Consumer	Commercial	Greenish	Attempted treatment
LDPE 4	Post-Industrial	Industrial	White	Washed and re-extruded
LDPE 5	Post-Industrial	Industrial	Black	Washed and re-extruded
LDPE 6	Post-Consumer	MSW (Yellow recycling bin), Spain	Black	Washed and re-extruded
HDPE 1	Virgin	Virgin	Transparent	None
HDPE 2	Post-Consumer	MSW (Yellow recycling bin), Germany	Greenish	Washed with hot water
HDPE 3	Post-Consumer	Bottle Flakes, Indonesia	Transparent	Washed
HDPE 4	Post-Consumer	MSW (Grey recycling bin)	Multi-colored	Washed
HDPE 5	Post-Consumer	MSW (Yellow recycling bin)	Multi-colored	Washed
HDPE 6	Post-Consumer	Recycled plastic from gasoline containers	Black	Crushed into flakes
PET 1	Virgin	Virgin	Transparent	None
PET 2	Post-Consumer	MSW (Yellow recycling bin), Spain	Bluish	Inlet (Before washing)^a^
PET 3	Post-Consumer	MSW (Yellow recycling bin), Spain	Bluish	Outlet (After washing)^a^
PET 4	Post-Consumer	Recycled PET 3D printer filament	Greenish	Unknown
PP 1	Virgin	Virgin	Transparent	None
PP 2	Post-Consumer	MSW (Yellow recycling bin), Germany	White	Washed with hot water
PP 3	Post-Consumer	MSW (Yellow recycling bin), Brazil	Green	Washed with cold water
PP 4	Post-Consumer	MSW (Yellow recycling bin), Brazil	Black	Washed with cold water

*MSW* = municipal solid waste.

^a^PET 2 and PET 3 are the same plastic, before and after washing process.

The collected plastic samples were meticulously processed to ensure homogeneity and consistency. Each plastic type underwent size reduction and homogenization through a Retsch SM 200 cutting mill (Haan, Germany) equipped with a 1.0 mm sieve. Fifty grams of each processed sample were collected and stored securely, awaiting subsequent analysis.

It is crucial to note that these carefully chosen plastic samples serve as representative models for understanding the intricacies of contamination and migration dynamics within the recycling landscape.

Note that all virgin plastic samples were obtained from specific suppliers dedicated to the production of plastic granules. These samples come directly from a standard production process and were not manufactured in our laboratories or sourced from commercial products. The recycled waste samples were sourced from various companies specializing in plastic recycling. These companies obtain plastic from diverse sources, including post-consumer, post-commercial, and post-industrial origins. The waste is then subjected to recycling processes to produce new granules. It is important to note that these samples were not produced or treated in our laboratories.

### Bisphenol A, S, F and M analysis

The extraction process of the plastic samples was performed in an ASE® 350 extractor (Accelerated Solvent Extraction). For sample extraction, pure ACN was used as solvent, supported by scientific evidence demonstrating its superiority in extracting the target compounds compared to alternatives such as methanol (MeOH) or ethyl acetate (ACE)^20^.

To perform the extraction, a small layer of diatomaceous earth (purchased from Thermo Fisher Scientific, Spain) is introduced into the extraction cell to be used as filler, followed by a cellulose extraction thimble, purchased from Fisher Scientific, Spain (Single thickness, size 22 mm × 80 mm) into which approximately 2 g of plastic are placed. Once the extraction cell is prepared, 5 µL of BPA-^13^C_12_ (100 ng/µL) purchased from Techno Spec, Barcelona, Spain, used as recovery/surrogate, are added, allowed to sit for half an hour, and the cell is further filled with diatomaceous earth before proceeding with the extraction. The extraction conditions used in the ASE® 350 are detailed in Table S1 of the supplementary material.

Upon completion of the extraction process, the extract is concentrated to a final volume of approximately 1 mL using a Super-Vap (Fluid Management Systems) purchased from Techno Spec, Barcelona, Spain, and finally diluted in Milli-Q water with the following specifications: resistivity 18.2 MΩ·cm (25 °C), TOC (Total Organic Carbon) < 5 ppb, particles/ml (< 0.22 µm) < 1, in a 9:1 ratio.

To purify the sample and remove impurities from the extract, samples were cleaned following an adaptation of the procedure proposed by Moid AlAmmari et al.^37^ .The extracts were eluted through C18 SPE cartridges purchased from Agilent (Spain), previously conditioned with 5 mL of ACN followed by 5 mL of Milli-Q water, at an elution rate of 1 mL/min. Once the columns were conditioned, the sample was passed through at a rate of 1 mL/min, and after the process was completed, the column was washed with a 9:1 solution of Milli-Q water and ACN and allowed to dry for 10 min. Finally, the analytes were eluted with 10 mL of pure ACN.

The final stage of the process involves reconcentrating the 10 mL of ACN containing the analytes using the Super-Vap to a final volume of approximately 1 mL and adding 5 µL of BPA-D_16_ (100 ng/µL), purchased from Techno Spec, Barcelona, Spain, used as a internal standard.

### UHPLC-MS/MS: method optimization

In this study, Ultra High-Performance Liquid Chromatography-Tandem Mass Spectrometry (UHPLC-MS/MS) (Agilent 1290 Infinity) with triple quadrupole mass spectrometry utilizing JetStream and iFunnel technology (QQQ-6490) was employed for bisphenols quantification.

The C18 column, characterized by 1.8 μm diameter particles and a length of 50 mm, demonstrated efficient separation of bisphenols within a reasonable timeframe. The selection of this column was based on its capability to separate all four bisphenols in under 15 min, yielding well-defined peaks without significant interferences.

Chromatography programming played a critical role in achieving efficient bisphenol separation. Liquid chromatography parameters were adjusted to enhance compound resolution. A mobile phase comprising a water and acetonitrile (ACN) mixture was chosen; a gradient elution program was established to improve compound resolution and ensure complete separation of the four bisphenols: ACN increased from 50 to 80% over 2 min, and it was maintained at 80% for 2.50 min to eliminate residues before returning to 50% for the next injection. A flow rate of 0.35 mL/min and a column temperature of 30 °C were utilized.

Configuring the mass spectrometer was another critical step in achieving efficient bisphenol detection. Multiple Reaction Monitoring (MRM) mode was employed to enhance selectivity and sensitivity. Two transitions were selected for each bisphenol, one for quantification and one for confirmation, with specific fragmentation parameters established for each transition. The LC and MS conditions for the analysis of BPA, BPA, BPF, and BPM by UHPLC-MS/MS are documented in Table S2 of the supplementary material, along with the MSMS parameters and collision energies, which can be referenced in Table S3 of the supplementary material.

### Migration of BPA, BPS, BPF and BPM to food simulants and ultrapure water

In the present study, experiments on migration of bisphenols were conducted using small fragments of shredded plastic (around 1 mm in size), equivalent to approximately 2 g of LDPE plastic material. The primary objective of these experiments was to assess the potential migration of compounds from the plastic material into food simulants and ultrapure water, with the aim of simulating food contact situations in plastic packaging.

To ensure accuracy in the quantification of the compounds of interest, an internal standard (BPA-^13^C_12_) was added to the sample prior to the start of the experiment. The plastic scraps were immersed in 20 mL of two different food simulants stipulated by Regulation (EU) No. 10/2011^8^ and ultrapure water (these simulants represent a variety of foods and beverages that plastic packaging may come into contact with under real-world conditions):

- Food Simulant A: 10% (v/v) ethanol solution in water.
- Food Simulant B: 3% (v/v) acetic acid solution in water.
- Food Simulant C: Ultrapure water.

The experiments were conducted over a period of 10 days at a temperature of 60 °C, simulating exposure times exceeding 30 days at room temperature or below, following the guidelines established by Regulation (EU) No. 10/2011^8^. Upon completion of the exposure period, the solutions were filtered to separate the liquid extract from the plastic material. This liquid extract underwent additional purification using C18 columns according to the aforementioned procedure. The quantification and analysis of BPA, BPS, BPF, and BPM were carried out using UHPLC-MS/MS.

All migration tests were carried out in duplicate, and the results were corrected by subtracting the obtained concentration of bisphenols for its concentration in blank samples, which were also analysed in duplicate.

### Treatment with polyethylene glycol

Polyethylene glycol 400 (PEG 400), sourced from Corquimia Industrial S.L. in Barcelona, Spain, was employed as agent for extracting the target contaminants. This study addressed the most heavily contaminated samples from each plastic type (LDPE, HDPE, PP, and PET), with one sample selected from each kind of plastic material.

A total of 25 g of these highly contaminated plastic samples, which are the original samples before extraction, underwent treatment in a 500 mL solution of PEG 400. These samples have already been recycled using existing technologies. After the extraction of 25 g of each plastic sample, the remaining 500 mL of PEG 400 solution is disposed of according to our laboratory's standard waste disposal procedures. This typically involves proper labelling and segregation of the waste solution for appropriate disposal in accordance with local regulations. Furthermore, it is important to clarify that the quantity of polyethylene glycol used for conducting the experiments is an excess amount, aimed at assessing the maximum potential for PEG removal, which does not imply that this PEG/plastic ratio is necessary or definitive.

The extraction was carried out at constant temperature of 120 °C and continuous agitation using a Magnetic Hotplate Stirrer Model VMS-C7 Advanced (purchased from VWR, Spain), equipped with precise temperature control, over a 2-h extraction period.

Following the completion of the extraction, the plastics were separated from the PEG 400 using a sieve. Any residual PEG adhering to the plastic surface was effectively eliminated through a meticulous rinsing procedure. This procedure entailed subjecting the plastics to a 30-min rinsing cycle using 500 mL of fresh water, with gentle agitation in a 1 L container employing a magnetic stirrer. Subsequently, the water was drained, and the treated recycled plastics were allowed to air dry for 48 h at ambient temperature.

Tables S4, S5 and S6 (Supplementary Materials) show the recoveries of the surrogate standards used for the analysis during the analysis of each sample, during migration tests and during PEG treatments respectively. Also limits of detection were calculated according to literature^38^, and the results are shown in Table S7.

### Supplementary Information

Supplementary Information.

## Data Availability

The datasets generated during and/or analyzed during the current study are available from the corresponding author on reasonable request.
