# Supplementary material for: Analysis and removal of bisphenols in recycled plastics using polyethylene glycol
Source: Sci Rep. 2024 Jun 4;14:12824. doi: 10.1038/s41598-024-63800-7 (PMC11150417; doi:10.1038/s41598-024-63800-7)
Supplement: Supplementary file 1 — Supplementary Information. [file 41598_2024_63800_MOESM1_ESM.docx]

**Supplementary material**

**Analysis and Removal of Bisphenols in Recycled Plastics Using Polyethylene Glycol**

Samuel S. Núñez^1,2^, Núria Ortuño^1,2^, Julia Moltó^1,2^, Sabrina Fernández-Durán^1^, Juan A. Conesa^1,2*^

^1^ Department of Chemical Engineering, University of Alicante, P.O. Box 99, E-03080 Alicante (Spain); diqui@ua.es

^2^ Institute of Chemical Process Engineering, University of Alicante, P.O. Box 99, E-03080 Alicante (Spain); iipq@ua.es

***** Correspondence: ja.conesa@ua.es; Tel.: +34-965903400

**The supplementary material contains 6 pages of additional information and includes 7 tables.**

**Table S1.** Extraction conditions in the ASE 350 system.

| **Extraction Conditions** | |
| --- | --- |
| Oven temperature: | 140 ºC |
| Pressure: | 1500 psi |
| Solvent: | Acetonitrile |
| Call Heat up Time: | 6 min |
| Static Time: | 3 min |
| Flush Volume: | 100% of cell volume |
| Static Cycles: | 3 |
| Purge Time: | 60 s |
| Total Volume: | 30 mL |
| Total Time: | 20 min |

**Table S2.** Instrument conditions for the analysis of BPA, BPS, BPF and BPM by UHPLC-MS/MS.

| **LC conditions** | |
| --- | --- |
| Instrument | Agilent 1290 Infinity UHPLC |
| Column | Agilent ZORBAX Eclipse Plus C18 2.1mm x 50mm, 1.8mm |
| Column temperature | 30º |
| Injection volume | 2 mL |
| Mobile phase | A) Water + 0.2mM NH_4_F |
|  | B) Acetonitrile + 0.2mM NH_4_F |
|  | Gradient |
|  | Time A (%) B (%) |
|  | 0 50 50 |
|  | 1,2 40 60 |
|  | 1,7 40 60 |
|  | 2 20 80 |
|  | Stop time 2.50 min |
|  | Post time 2 min |
| Flow rate | 0.35 mL/min |
| **MS conditions** | |
| Instrument | Triple Quadrupole Mass Spectrometer with  JetStream and iFunnel Technology (QQQ-6490) |
|  |  |
| ION mode | Negative ionization |
| Capillary voltage | 3.5 V |
| Drying gas (N_2_) | 11 L/min |
| Drying gas temperature | 250 ºC |
| Nebulizer | 45 psi |
| Source gas heater | 375 ºC |
| Source gas flow | 12 L/min |
| Nozzle voltage-Vcharging | 500 V |

**Table S3.** MS/MS parameters and collision energy for the analysis of BPA, BPS, BPF, and BPM.

| **Analyte** | **Precursor Ion  (m/z)** | **Product Ion (m/z)** | **Dwell time (ms)** | **Frag   (V)** | **Collision energy  (V)** | **Polarity** |
| --- | --- | --- | --- | --- | --- | --- |
| BPA | 227 | 211.1 | 50 | 380 | 30 | Negative |
|  |  | 133 |  |  | 26 |  |
| BPA-D16 | 241 | 142 | 50 | 380 | 20 | Negative |
|  |  | 97 |  |  | 20 |  |
| BPA-13C | 239 | 224 | 50 | 380 | 20 | Negative |
|  |  | 139 |  |  | 26 |  |
| BPS | 249 | 184 | 50 | 380 | 40 | Negative |
|  |  | 108 |  |  | 40 |  |
| BPF | 199 | 105 | 50 | 380 | 20 | Negative |
|  |  | 93 |  |  | 20 |  |
| BPM | 345 | 329 | 50 | 380 | 50 | Negative |
|  |  | 133 |  |  | 60 |  |

**Table S4**. Recoveries of the bisphenols analysis of each sample.

| **Sample** | **Recovery (%)  (BPA ^13^C_12_)** | **RSD** |  |
| --- | --- | --- | --- |
|  |  |  |  |
| LDPE 1 | 73% | 6% |  |
| LDPE 2 | 60% | 6% |  |
| LDPE 3 | 75% | 3% |  |
| LDPE 4 | 68% | 3% |  |
| LDPE 5 | 78% | 1% |  |
| LDPE 6 | 76% | 4% |  |
| HDPE 1 | 73% | 2% |  |
| HDPE 2 | 67% | 2% |  |
| HDPE 3 | 74% | 4% |  |
| HDPE 4 | 79% | 2% |  |
| HDPE 5 | 72% | 7% |  |
| HDPE 6 | 69% | 3% |  |
| PET 1 | 77% | 2% |  |
| PET 2 | 70% | 0% |  |
| PET 3 | 63% | 6% |  |
| PET 4 | 66% | 3% |  |
| PP 1 | 74% | 2% |  |
| PP 2 | 66% | 2% |  |
| PP 3 | 68% | 5% |  |
| PP 4 | 75% | 1% |  |
|  |  |  |  |
| MAX | 79,38% | |  |
| MIN | 59,68% | |  |

**Table S5.** Recoveries of the surrogate during migration tests.

| **Sample** | **Recovery (%)  (BPA ^13^C_12_)** | **RSD** |  |
| --- | --- | --- | --- |
|  |  |  |  |
| Simulant A | 67% | 11,11% |  |
| Simulant B | 77% | 0,21% |  |
| Ultrapure Water | 72% | 4,29% |  |
|  |  |  |  |
| MAX | 77% | |  |
| MIN | 67% | |  |

**Table S6**. Recoveries of the standards during PEG treatments.

| **Sample** | **Recovery (%)  (BPA ^13^C_12_)** | **RSD** |  |
| --- | --- | --- | --- |
|  |  |  |  |
| LDPE 6 | 79% | 9% |  |
| HDPE 4 | 56% | 8% |  |
| PP 4 | 75% | 1% |  |
| PET 4 | 85% | 1% |  |
|  |  |  |  |
| MAX | 85% | |  |
| MIN | 56% | |  |

**Table S7**. LOD and LOQ calculated.

| **Compound** | **Sb** | **m** | **LOD (ng/g)** | **LOQ (ng/g)** |
| --- | --- | --- | --- | --- |
| BPA | 0,835 | 22,41 | 0,056 | 0,186 |
| BPF | 0,436 | 22,56 | 0,031 | 0,103 |
| BPS | 2,138 | 571,88 | 0,006 | 0,019 |
| BPM | 0,518 | 58,72 | 0,013 | 0,044 |
